# Carbolic Acid and Its Therapeutical Uses

**Published:** 1869-07

**Authors:** 


### Selections.

Carbolic Acid and its Therapeutical Uses—A paper read before the Cincinnati Academy of Medicine June 7, 1869, by Wm. B. Davis, M. D.—Carbolic acid was discovered by Runge as far back as 1834, yet the attention of the medical profession was not directed to it as a remedial agent until quite recently. Now its use, both in medical and surgical practice, is varied and extensive.

Carbolic acid, strictly speaking, is not an acid, but an alcohol. It is distilled from coal tar, and when perfectly pure is a colorless crystaline mass, disposed to deliquescence. Of the crystal preparations found in commerce, Calverts of Manchester, is pure, and Mercks of Darmstadt, is 98 per cent. pure acid.

In many respects carbolic acid resembles creosote, and by some the two are considered identical, but Hlasiwetz in 1858, and Hugo Muller, in 1864, showed that creosote was a different body from carbolic cresylic acids. It dissolves in all proportions in Glycerine, alcohol, ether, acetic acid and the fixed oils. With twenty parts of water it forms a permanent emulsion.

Mr. Crooke's careful and extended experiments with it led him to the following conclusions, viz:

Carbolic Acid has but slight coagulating power on albumen. [Most writers differ with him on this point, claiming that it will coagulate the albuminous portions of the tissues whenever it comes in contact with them.]

It has no power of retarding oxidation.

It has scarcely any action on fœted gases; but it attacks the cause which produces them, and at the same time, puts the organic matter in such a state that it never re-acquires its tendency to purify.

It has a special action on the fermentation induced by organized matter; it not only arrests it instantly, when in progress, but it prevents the development of future fermentation.

It has no action on purely chemical ferments. It acts by attacking vitality in some mysterious way. The various infusoria of water, as well as small fish are instantly killed by a few drops of it. Fleas, moths, bugs and insect life generally, as well as animals as large as mice are destroyed by it.

The powerful action which carbolic acid exerts on the phenomena of life is the most remarbable property which it possesses. It may be looked upon as the test proper for distinguishing vital from purely physical phenomena, and in most cases its action is characterized by the certainty and definitness of a chemical re-agent. In the presence of it, the development of embryotic life is impossible, and before its powerful influence all minute forms of animal life must inevitably perish. (*Third Report of Commission on Cattle Plague*, p. 192, 193.)

As an antiseptic and disinfectant carbolic acid has no equal, 1-1000, even 1-5000 will prevent decomposition, fermentation or putrefaction of Blood, Urine, &c.

The sewers of London were kept perfectly sweet during the existence of cholera in 1866 by 1-10000 part.

In large doses it is a dangerous poison. The recent journals, both of America and Europe, report several deaths occasioned by it.

Prof. J. G. Pinkham pronounced it a poison not inferior to oxalic acid, and hardly so to strychnine. It is rapidly absorbed by the system and rapidly eliminated from it, chiefly by the kidneys.

The local action of the poison is that of a caustic, irritant and sedative. The general action is that of a powerful neurotic, causing trembling, convulsions, giddiness, headache, insensibility, a cold clammy surface, a feeble intermittent rapid pulse, great prostration and death.

In treatment, the chief reliance must be placed upon measures of evacuation and stimulation. (*Medical and Surgical Reporter*, *December*, 1808.

If M. Pasteur's theory be correct, that all fermentation and putrefaction depends upon the living germs which the air contains, we have in carbolic acid an agent whose special action is directed against vitality, and whose fumes will destroy all organic and organized bodies, which the air may bring with them.

Dr. Argus Smith says it may be considered absolutely certain, that all organic substance, whether of the nature of plague or any other disease, will be arrested in their course of activity by it.

Mr. Crookes has proven that it will destroy the contagion of cattle plague and the virus of vaccine, and that cattle are perfectly protected by it from the contagion of plague. His detailed experiments "point forcibly to the possible prevention and cure of all zymotic diseases which attack the human race. Every argument brought forward, every experiment detailed and every result obtained of this investigation apply with overwhelming force to such visitations as typhus and typhoid fever, small pox, diphtheria and cholera." (*Page* 201, *Cattle Plague Report.*)

These conclusions agree with those of Dr. Jules Lemaire, whose work on phenic acid was published in Paris in 1865. M. Lemaire shows that carbolic acid is the most powerful acknowledged means of contending with contagions and pestilential diseases, such as cholera, typhus fever, small pox, &c.

Dr. A. E. Sansom read a paper before "Medical Society of London," April 5, 1869, in which he gave a sketch of a history of the theory of fermentation in its relation with zymotic disease. He claimed that the analogy between fevers and fermentation has been taught since the earliest days of physic, and showed that the investigation of the real nature of fermentation has thrown much light on the subject by proving that living molecules were the prime causes of the process. The author considered that the potential energy of the morbific molecule as well as of the ferment could be stored up in no inorganic material—this must possess power of vitality. He divided the germs of disease into two classes, according as they multiply (a) in the blood, (b) in the intestimal canal. He showed by experiment that fermentation can take place in the gastro-intestinal tract of mice. He then discussed the means of destroying these germs and considered that the most powerful agent with which we are acquainted is carbolic acid. His experience, fortified by that of others, was that they relieved dyspepsia, checked pus formation, and seemed to diminish the intensity of the symptoms of zymotic disease. (*Medical Times and Gazette, April* 24, 1869.)

It is shown that carbolic acid is destructive to all embryotic as well as all minute forms of animal life, and it is proven that in certain doses it is equally destructive to human life. Will the dose which may be required to destroy the virus of disease endanger the life of the patient? Dr. Angus Smith believes there is an amount which will destroy the germs of disease and not destroy life. On page 163 of his Report (Cattle Plague Com.,) he states: "I have heard of no one being injured by breathing air, scented by carbolic acid all day for a long time together, and I have myself breathed it night and day in a mild state—very strong it must never be breathed. Flesh will absorb carbolic acid and become so saturated that when roasted it will cease to smell like flesh. I am informed that men working in equally strong vapor are not injured."

Mr. Crookes says that medical and scientific writers were unanimous in the opinion that small internal doses of carbolic acid were attended with no injurious effect, and he gave it to cattle in such doses that their breath smelled of it for some hours without any injurious effects.

The internal administration of carbolic acid as a remedial agent in the treatment of disease, is just attracting the attention of the profession, and, consequently, there is not sufficient evidence to authoritatively state the exact quantity that will constitute a proper dose. Prof. Lionel Beale thinks that a solution of one part carbolic acid to 200 parts water is as strong as should be used. Dr. H. W. Fuller, of St. George's Hospital, says that "as far as the mere dose was concerned, I found that some adults—especially men who have been spirit drinkers—could take ten or twelve minims without inconvenience, and notwithstanding the occurrence of a certain degree of discomfort, could take doses of fifteen minims three or four times a day for many days consecutively; but that most persons, especially women, began to complain when the dose had been increased to eight or ten minims and found six or seven minims a full dose."

In the State Lunatic Asylum at Utica, N. Y, the standard solution is one grain to the ounce of water, and the dose of this solution is a drachm. I have administered carbolic acid internally since September 1866, and I have been in the habit of ordering four grains to the ounce of water, and of this solution I gave two drachms every three or four hours to adults and one drachm to children; and I have never known any injurious effects result from its use in this strength.

Carbolic acid is now extensively used in surgery. Prof. Joseph Lister, of Glasgow, has achieved a world-wide reputation by his surgical uses of this agent. Adopting the germ theory, he confidently applied carbolic acid in compound and comminuted fractures, wounds of joints, acute and chronic abscesses, tumors and wounds generally, and with the remarkable result of immediately converting compound fractures into simple fractures with superficial sores, the arrest of deep-seated suppurations and the prevention of constitutional disturbance, &c. [The details of his treatment can be found in the *London Lancet*, March, 1867.]

Prof. James Syme and other distinguished surgeons have adopted Mr. Lister's mode of treatment and with the most favorable results. Mr. McCormac in the *Dublin Quarterly*, February, 1869, details eight cases, and Joseph Bell, in the *Edinburgh Medical Journal*, May, 1869, nine cases treated according to Mr. Lister's antiseptic method and with the most astonishing results. Aladdin with his lamp performed nothing more wonderful nor half so satisfactory, as carbolic acid did in most of these cases.

Combined with linseed oil in proportion of 1 to 10, carbolic acid in burns arrests pain, prevents suppuration, dries up the bullæ and effects a speedy cure.

It is used in the treatment of syphilitic sores (primary and secondary.) "Among a large number of patients with primary sores, those who had used carbolic acid lotion have been freer from buboes. The sores have healed, and induration disappeared more rapidly than with those who had not the lotion.

There is strong reason to believe that the occurrence of secondary symptoms is less frequent, *cœteris paribus*, among those using the lotion. (*London Lancet*, *page* 217, 1869.)

It is especially useful in those forms of skin disease depending on parasites or accompanied by the development of any of the forms of fungi.

All parasites which have their habitat, in or on man, find in carbolic acid an uncompromising foe, and it is equally destructive to the vermin which infest some houses, such as roaches, bedbugs, &c.

In the treatment of gonorrhœa ozæna, otorrhœa ulcerated sore throat, &c., it has been found efficacious. Internally it is administered in the treatment of phthisis pulmonalis, pneumonia, bronchitis, particularly when the sputa is profuse, offensive or purulent, typhus and typhoid fever, measles, scarlet fever, as well as in dyspepsia, diarrhœa and vomiting.

As a prophylaxis of scarlet fever it has been used by Mr. Amos Beardsley. When a patient suffers from scarlatina, he is washed all over, once or twice a day, with diluted carbolic acid, one drachm to a pint. Mr. Beardsley says that in no case in which he has tried it with the first case in a house has there been any further spread of Scarlatina in the family. He has now so much experience as to be convinced that this plan is most useful in preventing the emanation of contagious influence from patients. (*Practitioner, February*, 1869.)

My first experience with the internal administration of carbolic acid was in 1866 in the treatment of diptheria. The following case, treated in that year, I select from a number, because of its typical character and severity.

Luther J——, aged 4 years, was observed to droop for two or three days prior to the night of October 20, 1866, when he was suddenly seized with convulsions. I was immediately sent for, but he recovered his consciousness before my arrival. I found him with a hot skin, flushed face, bounding pulse, breathing labored, tongue furred, submaxillary glands enlarged, tonsils swollen, urine albuminurious. Ulceration of the tonsils set in on the second day and by the 24th inst. a black, slough-like covering formed on the tonsils, which rendered the breath very fœtid. His strength rapidly failed and my hopes for his recovery were but slight until the morning of the 29th inst., when some improvement was manifested. From this date he slowly convalesced until his full recovery in the latter part of December. When convalescence had been well established, so that he was no longer confined to his bed, his mother surprised me one morning as I entered, by exclaiming: "Doctor, my child is cross-eyed!" An examination confirmed this statement. With strabismus there was defective vision, owing to loss of adjusting power. There was also paralysis of the faucial muscles, which impaired the voice. The muscles of the back and neck were likewise affected, so that he could not stand erect or properly support his head.

My treatment during the first stage was ½ of a grain of carbolic acid in conjunction with chlorate of potash, every 3 hours, and a wash for the tonsils of carbolic acid, 5 grains to the ounce of water. During convalescence, muriated tinct. of iron, quinine, brandy and beef tea were given. I have never known a case as severe as this one to recover on any other treatment. I have continued the use of the acid in the treatment of diphtheria and with very satisfactory results.

I have treated 20 cases of measles with it—of this number 17 recovered and 3 died. The latter were cutting teeth at the time, and convulsions supervened, of which they died.

I have administered it to 17 cases of scarlet fever and lost 5 of them; these five were malignant ones; 3 died in one room and 2 in another. I think they would not have recovered under any treatment, yet I do not think I gave carbolic acid as fair a test in these cases as it deserved. In the future I will put malignant cases in separate rooms, disinfect the premises and have the air they breathe slightly charged with carbolic acid.

I have administered it to fourteen cases of small pox and all recovered but one Five were confluent and nine were well marked, distinct cases. Three of the five confluent ones had been vaccinated, and of this number was the one who died. Of the nine distinct cases, four had been vaccinated and five had not. The secondary fever in several was entirely wanting, and very mild in those who had any.

The most remarkable result observed was, that not one of the thirteen who recovered was disfigured by pitting. I recently visited two of the patients who had passed through the severest form of confluent small pox. One was a woman aged 58 years, who had been vaccinated in her youth; the other a girl of 7 years, who had never been vaccinated. In the first case three months had elapsed since her recovery; in the other, two months. Upon a close scrutiny I could discover some superficial pitting on the nose and forehead, but a few feet distant they were not observable. There was no disfiguration. My general prescription in the treatment of these cases was as follows:

℞.—Crystalized Carbolic Acid, grs. xvi.
Chlorate of Potash, ʒii.
Spir. Nit. Dulc. ℥i.
Syrup Ipecac ℥i.
Syrup Tolu ℥i.
Glycerine ℥i. M.

S.—½ tablespoonful every three hours during the eruptive fever.

A lotion of carbolic acid, grs. x, to glycerine ℥j, was applied to the face and hands.

Dr. Cassat, of this city, has treated four cases of gonorrhœa with carbolic acid in the strength of twenty grains to the ounce of water, used as an injection. And in each case the discharge was arrested within the first twenty-four hours and did not return.

I have used it in two cases. In the first one I directed four grains to the ounce of water, but it did not control the discharge, although the patient injected it three times per day for a week. In my second case I ordered a solution of twenty grains to the ounce, and the patient injected it twice and the discharge stopped and did not return. No other medication was used. In the latter strength it produced severe pain, which continued from one to three hours.

I treated a patient who was very much broken down with a carbuncle, full six inches in diameter. I made a crucial incision, washed it three times per day with a lotion of four grains of carbolic acid to the ounce, and dressed it with a salve containing four grains of the acid to the ounce and administered the acid internally in conjunction with tonics. The patient made a rapid recovery and was able to resume his work in three weeks.

Dr. John Davis first directed my attention to the internal use of carbolic acid, and he has kindly furnished me the subjoined record:

The first instance of my administering carbolic acid internally was on the 25th of September, 1866. I was then called for the first time to see Mrs. J.——, living near Cincinnati. She had been suffering with severe cough for several weeks, and was far gone in pregnancy. Upon entering her room I perceived a very offensive odor of putrescent animal matter. Upon nearing her this stench was more intense, and her sputa, from whence this odor proceeded, affected my olfactories almost unbearably. They were rust colored and copious, and her right lung presented dullness to percussion over the whole extent of the lower two-thirds of its surface. She was much emaciated and had but little appetite.

I ordered as follows:

℞ —Crystalized Carbolic Acid, gtt. xvi.
Chlorate of Potassa, ʒi.
Sulphate of Morphia, grs. ii.
Syrup of Tolu, ℥j.
Peppermint Water, ℥iii. M.

S.—Half a tablespoonful every three hours while awake, and the right side of the chest to be painted over once per day with a mixture composed of equal parts of Tinct. of Iodine and Alcohol. Half a tablespoonful of Huxam's Tinct. of Bark was also ordered to be given three times per day.

The next day the stench which, on the day before filled her room, was now hardly perceptible, and in twenty-four hours more it had entirely disappeared to return no more.

Oct. 6.—This patient is now nearly well. She has but little cough, the dullness to percussion over her right lung has nearly disappeared and her flesh and strength are much what would be expected of any one very near the time of her confinement. Her recovery has been so rapid that I have made to her in all only seven visits, including that of this date.

Oct. 17.—She was delivered of a healthy child on the 7th inst, and is now quite well.

What led me to think of administering carbolic acid internally for this patient, was the horrible putrescent stench emanating from her. I knew that it had been proved that meat saturated with carbolic acid would undergo no decomposition. I therefore inferred that its influence on a decomposing vital animal organization might prove also effective. My success in this case led me immediately to using the acid as an antiseptic in almost all the cases of septic or zymotic diseases which I have been called to treat from that time to the present.

In diphtheria and typhoid fever I have found its use attended with remarkable success; also, in scarlet fever and measles.

In some cachectic conditions I have also found it to have a very good effect, as in the third stage of phthisis pulmonalis, to retard the destruction of tissue.

The following is from C. P. Brent, M. D., physician to the Hamilton County Jail:

George H——, aged 7 years, 42 Elm street, was attacked with Pneumonia, December 6, 1866. I treated the case in my usual way, but there was no cessation of the morbid action; the case passed through the first and second stages and into the third, resulting in suppuration of the lower lobe of right lung. The patient was gradually sinking, although tonics and stimulants were freely administered. The odor proceeding from his sputa was unbearable, and the whole room was filled with the sickening smell. The family and myself were daily expecting him to die, when, at the suggestion of Dr. John Davis, I gave him carbolic acid and in twenty-four hours there was a marked diminution in the odor, and in a few days it was scarcely perceptible. The patient began to improve rapidly and in a few weeks was a positive convalescent. He is now active and well.

I have used carbolic acid in another case of pneumonia, where there was suppurative action with the same good result. Also in a case of phthisis, where the breath and sputa almost drove the friends from the room, the offensive odor was entirely removed by its use.

I used carbolic acid very freely in the Hamilton County Jail in various affections, especially syphilis, all forms of ulcers, &c. I could not dispense with it any better than with the old standard remedies.

